# Acute uveitis: A rare adverse effect of miltefosine in the treatment of post-kala-azar dermal leishmaniasis

**DOI:** 10.1590/0037-8682-0208-2020

**Published:** 2020-12-21

**Authors:** Krishna Pandey, Biplab Pal, Roshan Kamal Topno, Chandra Shekhar Lal, Vidya Nand Rabi Das, Pradeep Das

**Affiliations:** 1 Indian Council of Medical Research - Rajendra Memorial Research Institute of Medical Sciences, Department of Clinical Medicine, Agamkuan, Patna, Bihar, India.; 2 Lovely Professional University, Department of Pharmacology, Jalandhar - Delhi G.T. Road, Phagwara, Punjab, India.; 3 Indian Council of Medical Research - Rajendra Memorial Research Institute of Medical Sciences, Department of Epidemiology, Agamkuan, Patna, Bihar, India.; 4 Indian Council of Medical Research - Rajendra Memorial Research Institute of Medical Sciences, Department of Biochemistry, Agamkuan, Patna, Bihar, India.; 5 Indian Council of Medical Research - Rajendra Memorial Research Institute of Medical Sciences, Department of Molecular Biology, Agamkuan, Patna, Bihar, India.

**Keywords:** Miltefosine, Post-kala-azar dermal leishmaniasis (PKDL), Acute uveitis

## Abstract

Post-kala-azar dermal leishmaniasis is a skin disorder occurring in 5-10% of visceral leishmaniasis patients after treatment with miltefosine,the first-line drug for this skin disorder. We reported a case of acute anterior uveitis,a rare adverse effect, experienced by a patient treated with miltefosine for post-kala-azar dermal leishmaniasis. This adverse effect developed after 15 days of miltefosine consumption, and the patient himself discontinued the treatment. The ophthalmic complication was completely resolved with antibiotics and steroid eye drops. After recovery from the ophthalmic complication, the patient was successfully treated with liposomal amphotericin B for the skin lesions.

## INTRODUCTION

Post-kala-azar dermal leishmaniasis (PKDL) is a vector-borne disorder affecting the surface of the skin. Reportedly, it occurs after the treatment of visceral leishmaniasis (VL) in 5-10% of cases^1^. The disease initially manifests as macular lesions that progressively develop into papular and nodular lesions. PKDL is not a systemic illness and does not interfere with the daily routine activity of the affected individual. Hence, patients do not seek treatment immediately. However, rapid treatment of these cases is vital for the elimination of kala-azar from the Indian subcontinent, as PKDL patients act as reservoirs of the parasites. There are very few drugs available for the management of PKDL, which include miltefosine, amphotericin B deoxycholate, and liposomal amphotericin B. All these drugs need to be administered long-term and may be associated with toxicities. Currently, miltefosine is being used as the first-line therapy for the treatment of PKDL in the Indian subcontinent. The most common adverse events associated with this therapy are gastrointestinal disturbances, such as nausea, vomiting, abdominal pain, and diarrhea^2^. Retinal degeneration with miltefosine has been reported in animal models^3^, butthere is limited data regarding its side effects in humans. However, two different case series studies from Bangladesh and India have reported the incidence of ophthalmic complications with miltefosine therapy^4^ ^,^ ^5^.

## CASE REPORT

A 42-year-old man from Chhapra, Bihar, visited the outpatient department of Rajendra Memorial Research Institute of Medical Sciences (RMRIMS), Indian Council of Medical Research, Patna, on December 10, 2018, with non-itching nodular eruptions on his face, hands, and abdomen. The patient had a history of VL 8 months back. On physical examination, the patient was afebrile and non-anemic, with no hepatosplenomegaly, and skin lesions with intact sensitivity. On investigation, his rK39 strip test was positive. Hence, PKDL was suspected,and the patient was advised to undergo skin smear microscopy and quantitative polymerase chain reaction (qPCR) analysis. His skin smear report was positive for Leishman-donovan bodies, and the parasite load for qPCR was 6723 parasites/µg of DNA. Hence, the patient was diagnosed with PKDL. 

**FIGURE 1:**
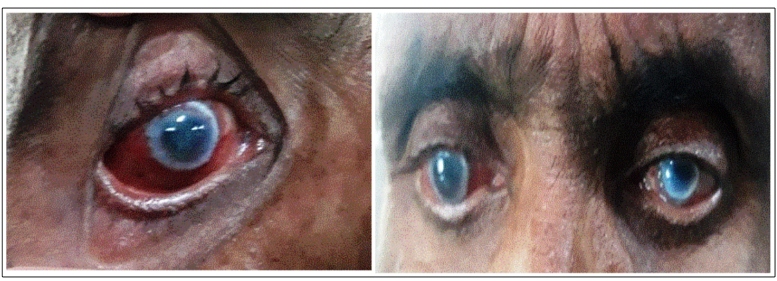
Bilateral cilliary congestion observed in both eyes of the patient,presumed as miltefosine-associated acute bilateral anterior uveitis

The patient was prescribed 100 mg of oral miltefosine capsules daily for 28 days. After 15 days of miltefosine consumption, he developed blurred vision, redness, photophobia, and foreign body sensation in both eyes (Figure 1). He stopped taking miltefosine on his own, but his symptoms did not improve. Six days later, he visited RMRIMS, Patna. The patient was referred to a higher ophthalmological center for consultation by a trained ophthalmologist. His ophthalmic investigation revealed best corrected visual acuity (BCVA) in the right and left eyeof 6/9 and 6/18, respectively. There was ciliary congestion in both eyes with no mucopurulent discharge. Anterior chamber examination revealed cells and flares in the anterior chamber along with fine keratic precipitates over the endothelium in both eyes. There was dense senile arcus in both eyes and mild posterior subcapsular cataract in the left eye. Posterior segment examination revealed no macular edema on +90D examination in either eye. Optical coherence tomography was not performed as the +90D examination showed no macular edema. Indirect ophthalmoscopy examinations were normal in both eyes. Routine laboratory examinations, including total blood cell count, differential white blood cell count, erythrocyte sedimentation rate, chest radiography, Mantoux test, venereal disease research laboratory test, and antinuclear antibody test were performed to exclude other common causes of uveitis. The results were insignificant to establish any systemic association for uveitis. Therefore, a presumptive diagnosis of acute anterior non-granulomatous uveitis was made, and the routine treatment for typical anterior uveitis was started. He was treated with topical antibiotics and steroid eye drops. After 3 weeks of treatment, his ophthalmological examination revealed resolution of the uveitis. BCVA was recorded as 6/6 and 6/12 in the right and left eye, respectively. No cells or flare was present in the anterior segment examination. Posterior segment examination was normal on +90D and indirect ophthalmoscopy examination. The patient was administered 30 mg/kg of liposomal amphotericin B (6 doses of 5 mg/kg twice weekly for 3 weeks) in 5% dextrose intravenously. Six months post-treatment, all the skin lesions disappeared and the qPCR report showed negligible parasites.

## DISCUSSION

Miltefosine is an alkylphosphocholine drug used for the treatment of breast carcinoma in the topical formulation^6^. In 2002, miltefosine was approved for the treatment of VL cases^6^. Owing to its excellent safety and effectiveness, it has been used as a first-line therapy for PKDL cases in India. It is the only orally available drug for leishmaniasis, and the recommended dose for VL is 2.5mg/kg/day (children) or 100mg/day (adults) for 28 days and 12 weeks for PKDL. Along with long-term therapy, it has a long terminal half-life (31 days). Dose- and time-dependent degeneration of the retina, mainly affecting the pigmented epithelium, was observed in rat models receiving miltefosine^3^. However, these changes were reversible after rechallenge therapy. Miltefosine requires administration for a long period (12 weeks) for its effectiveness in PKDL cases. The drug has many different mechanisms of action. First, apoptosis has been observed in *L. donovani* promastigotes^7^. Miltefosine also inhibits the synthesis of phosphatidyl choline, which is an important cell membrane component^7^. Another important mechanism of this drug is its cationic and amphiphilic properties, in which the drug binds to phospholipids and phosphocholine derivatives in the cell membrane of the parasite^7^. The membrane-soluble properties of the drug allow it to cross the cell membrane easily and accumulate inside lysosomes. This mechanism is probably responsible for the ophthalmic complications of miltefosine. Another proposed mechanism, as reported by Suman et al., is that the pKa and pH of the phosphoryl group in miltefosine are approximately 2 and 4.5-5, respectively. Hence, in the lysosomal environment, it may not be acidic enough to lead to protonation of the negativelycharged phosphoryl group and subsequent accumulation upon gaining polarity^5^ ^,^ ^7^ ^,^ ^8^.

Further studies are required to understand the mechanism of the ophthalmic complications of this drug. In our study, the patient was successfully treated with liposomal amphotericin B for the PKDL lesions. It is the second choice for the treatment of PKDL after miltefosine in India, and it is associated with fewer side effects. Patients experiencing similar complications as reported in other studies were also successfully treated with liposomal amphotericin B for their PKDL lesions^4^ ^,^ ^5^. In our case, ophthalmic complications developed within 2 weeks of the initiation of therapy. In another study, one patient developed complications within 2 weeks, and three patients developed complications after7-10 weeks of therapy^5^. Similar to our case, in another study, the therapy was stopped immediately^5^. We have treated more than 1,000 PKDL patients, but this is the first incidence of miltefosine-induced ophthalmic complications at our center. 

Although miltefosine is the first-line drug for PKDL, this emerging complication may limit its application. We recommend that patients with PKDL be counseled regarding this complication while dispensing miltefosine, so that this reaction can be minimized at initiation and untoward morbidity can be avoided. In addition, continuous monitoring of this complication is recommended in patients taking this medication.
